# PD01 - Respiratory allergens in human milk: potential impact on susceptibility to allergic airway disease

**DOI:** 10.1186/2045-7022-4-S1-P1

**Published:** 2014-02-28

**Authors:** Patricia Macchiaverni, Akila Rekima, Mathilde Turfkruyer, Laurent Mascarell, Sabi Airouche, Philippe Moingeon, Karine Adel-Patient, Antonio Condino-Neto, Isabella Annesi-Maesano, Susan L Prescott, Meri K Tulic, Valérie Verhasselt

**Affiliations:** 1 Institute of Biomedical Sciences, University of São Paulo, São Paulo, Brazil; 2 EA 6302 "Tolérance Immunitaire", Université de Nice Sophia-Antipolis, Hôpital de l'Archet, Nice, France; 3 Research and Development, Stallergenes SA, Antony, France; 4 INRA, UR496 Immuno-Allergie Alimentaire, CEA/IBiTeC-S/SPI, CEA de Saclay, F-91191 Gif sur Yvette cedex, France; 5 EPAR UMR-S 707 INSERM, France; 6 EPAR UMR-S 707 UPMC Paris6, Medical School Saint-Antoine, Paris, France; 7 School of Pediatrics and Child Health, University of Western Australia, Perth, Australia; 8 The International Inflammation “in-FLAME” Network, Worldwide Universities Network (WUN

## Background

Impact of exposure to environmental allergens during early life on allergic sensitization and disease development is controversial.

## Objective

We investigated whether airborne allergen from *Dermatophagoides pteronyssinus* (Der p), a major cause of allergic asthma, is found in human breast-milk and examined its impact on allergic outcome in a mouse model.

## Methods

Der p 1 was quantified in human colostrum and milk samples from Brasil, Australia and France by ELISA. Basophil degranulation assay was used to confirm immunogenicity of Der p. BALB/c mice were fostered by mothers exposed to Der p during lactation. Progeny allergic response to Der p was measured at 6-weeks.

## Results

Der p 1 was present in 58% Brazilian, 70% French, and 78% Australian colostrum. Median [Der p 1] was similar between countries (96 pg/mL). In mature milk, Der p1 was found in 55% of samples, median [Der p 1] was 65·9 pg/mL and was significantly lower than in colostrum (p=0·0001). Der p 1-containing milks were able to induce basophils degranulation. Mice breastfed by Der p-exposed mothers had 5-fold increased levels of Der p specific IgE and IgG1 compared to mice breastfed by naïve mothers. Their allergic airway inflammation was not affected.

## Conclusion

Early life exposure to ubiquitous respiratory allergens can take place through breastfeeding. An animal model mimicking the human situation shows early life exposure to Der p through milk primes the immune system. The presence of respiratory allergens in breast-milk may be an important factor in driving the early immune function towards allergic disease.

